# Supplementary material for: Insomnia symptoms and biomarkers of monocyte activation, systemic inflammation, and coagulation in HIV: Veterans Aging Cohort Study
Source: PLoS One. 2021 Feb 9;16(2):e0246073. doi: 10.1371/journal.pone.0246073 (PMC7872271; doi:10.1371/journal.pone.0246073)
Supplement: Attachment — Submitted filename: Response to Reviewers 2.docx [file pone.0246073.s001.docx]

PONE-D-20-30184

Insomnia symptoms and biomarkers of monocyte activation, systemic inflammation, and coagulation in HIV: Veterans Aging Cohort Study

PLOS ONE

**Additional Requirements**

**1. Please ensure that your manuscript meets PLOS ONE's style requirements, including those for file naming. The PLOS ONE style templates can be found at**

**https://journals.plos.org/plosone/s/file?id=wjVg/PLOSOne_formatting_sample_main_body.pdf and**

**https://journals.plos.org/plosone/s/file?id=ba62/PLOSOne_formatting_sample_title_authors_affiliations.pdf**

We have reviewed our revised manuscript and believe we made all necessary changes to align with the PLOS ONE journal style requirements as delineated in the file labeled “Revised Manuscript with Track Changes”.

**2. Please amend your current ethics statement to include the full name of the ethics committee/institutional review board(s) that approved your specific study.**

**3. Please provide additional details regarding participant consent. In the ethics statement in the Methods and online submission information, please ensure that you have specified (1) whether consent was informed and (2) what type you obtained (for instance, written or verbal, and if verbal, how it was documented and witnessed). If you are reporting a retrospective study of medical records or archived samples, please ensure that you have discussed whether all data were fully anonymized before you accessed them and/or whether the IRB or ethics committee waived the requirement for informed consent. If patients provided informed written consent to have data from their medical records used in research, please include this information.**

We have amended the Ethics Statement to include the requested details in red font. This statement has also been updated in the revised manuscript Methods section (pp. 4-5) and in the online submission form:

- VACS-9 is a prospective, multisite, cohort study of HIV-positive veterans and age, sex, race/ethnicity, and clinical site-matched HIV-negative veterans from nine Department of Veterans Affairs (VA) medical centers across the U.S. [29, 30]. The institutional review boards of the West Haven VAMC, the VAMC Tennessee Valley Health Care System, and the Vanderbilt University Medical Center IRB approved this study. All participants in the VACS 9 completed a written informed consent. All participants provided written informed consent to have their data from their medical records used for research.

**4. We note that you received funding from a commercial source: ViiV Healthcare, Gilead Sciences, GlaxoSmithKline, Bristol-Myers Squibb, General Electric, Merck.**

**Please provide an amended Competing Interests Statement that explicitly states these commercial funders, along with any other relevant declarations relating to employment, consultancy, patents, products in development, marketed products, etc.**

**Within this Competing Interests Statement, please confirm that this does not alter your adherence to all PLOS ONE policies on sharing data and materials by including the following statement: "This does not alter our adherence to PLOS ONE policies on sharing data and materials.” (as detailed online in our guide for authors http://journals.plos.org/plosone/s/competing-interests). If there are restrictions on sharing of data and/or materials, please state these. Please note that we cannot proceed with consideration of your article until this information has been declared.**

We have amended the Competing Interests Statements with the requested information in red font. This statement has also been provided in the revised cover letter.

- The authors report the following potential competing interests related to commercial sources. Dr. Bedimo reports funding from ViiV Healthcare and service on scientific advisory boards for Merck & Co, and ViiV Healthcare. Dr. Gupta reports funding from Gilead Sciences, travel support from Gilead Sciences and Bristol-Myers Squibb, and receipt of advisory board fees from Gilead Sciences and GlaxoSmithKline/ViiV Healthcare. Dr. Budoff reports funding from General Electric. Dr. Butt reports funding from Gilead Sciences. These commercial sources did not play any role in the study design, data collection and analysis, decision to publish, or preparation of the manuscript and does not alter our adherence to PLOS ONE policies on sharing data and materials. Our restrictions on sharing of data is due to US Department of Veterans Affairs regulations (see Data Availability statement).

We apologize for our confusion in reporting applicable funding for competing interests and thank you for the opportunity to correct this issue. Our manuscript under review with PLOS One was initiated in 2017. Upon further clarification, Dr. Butt reported funding from Gilead Sciences from 2015-2018 but noted his prior funding from Merck was from 2015-2016. Thus, we only list Dr. Butt’s funding from Gilead Sciences in the Competing Interest Statement. I have made the correct to the above statement here as well as the copy provided in the cover letter.

**5. We note that you have indicated that data from this study are available upon request. PLOS only allows data to be available upon request if there are legal or ethical restrictions on sharing data publicly.**

**In your revised cover letter, please address the following prompts:**

**a) If there are ethical or legal restrictions on sharing a de-identified data set, please explain them in detail (e.g., data contain potentially sensitive information, data are owned by a third-party organization, etc.) and who has imposed them (e.g., an ethics committee). Please also provide contact information for a data access committee, ethics committee, or other institutional body to which data requests may be sent.**

**b) If there are no restrictions, please upload the minimal anonymized data set necessary to replicate your study findings as either Supporting Information files or to a stable, public repository and provide us with the relevant URLs, DOIs, or accession numbers.**

We have amended the Data Availability Statement to include additional detail in red font. This statement is also now included in the revised cover letter:

- Due to US Department of Veterans Affairs (VA) regulations and our ethics agreements, the analytic data sets used for this study are not permitted to leave the VA firewall without a Data Use Agreement. This limitation is consistent with other studies based on VA data. However, VA data are made freely available to researchers with an approved VA study protocol. All data are anonymized prior to being provided to researchers outside of the immediate VA research team. For more information, please visit https://www.virec.research.va.gov or contact the VA Information Resource Center at VIReC@va.gov.

**Review Comments to the Author**

**Reviewer #1: In this study, performed by Brittanny Polanka and co - workers among 1,542 people with HIV from the Veterans Aging Cohort Study Biomarker Cohort the authors carried out a thorough analysis with multiple variables, which allowed them to reach significant results concerning the lack of associations between insomnia symptoms and higher circulating levels of markers of monocyte activation, systemic inflammation, and coagulation in these patients.**

**Reviewer #2: PLWH have many co-morbidities that have yet to be fully mechanized and this is an important field to which researchers can contribute. The authors of the manuscript were interested in understanding whether insomnia is a risk factor in HIV cardiovascular disease and modeled insomnia data from the VACS Survey with various compounding risk factors to allow for proper adjustments. Unfortunately, their data did not support their hypothesis. Nonetheless, the authors presented their negative data with gusto and this reviewer applauds their discussion section, where the authors dove into potential reasons for why their hypothesis was not supported and other published data with mixed results.**

**Please revise people with human immunodeficiency virus (PWH) to people living with human immunodeficiency virus (PLWH) throughout the manuscript.**

**Correct line markers 113-118 overlapping with Table 1.**

**Line 251: “our samples high prevalence” should be “our sample’s high prevalence”**

Thank you for identifying these 3 items for correction. We now refer to the HIV population as people living with HIV (PLWH) throughout the manuscript and have made the identified corrections to Table 1 and line 241.
